# Brazilian transgender children and adolescents: Attributes associated with quality of life*

**DOI:** 10.1590/1518-8345.3504.3351

**Published:** 2020-11-06

**Authors:** Fernanda Karla Nascimento, Roberta Alvarenga Reis, Alexandre Saadeh, Fran Demétrio, Ivaneide Leal Ataide Rodrigues, Sueli Aparecida Frari Galera, Claudia Benedita dos Santos

**Affiliations:** 1 Universidade de São Paulo Escola de Enfermagem de Ribeirão Preto, PAHO/WHO Collaborating Centre for Nursing Research Development, Ribeirão Preto, SP, Brazil.; 2 Scholarship holder at the Coordenação de Aperfeiçoamento de Pessoal de Nível Superior (CAPES), Brazil.; 3 Universidade Federal do Rio Grande do Sul, Faculdade de Odontologia, Porto Alegre, RS, Brazil.; 4 Universidade de São Paulo, Faculdade de Medicina, Instituto de Psiquiatria, Hospital das Clínicas, São Paulo, SP, Brazil.; 5 Pontifícia Universidade Católica de São Paulo, Faculdade de Ciências Humanas e da Saúde, Campus Monte Alegre, São Paulo, SP, Brazil.; 6 Universidade Federal do Recôncavo da Bahia, Centro de Ciência da Saúde, Cajueiro, BA, Brazil.; 7 Universidade do Estado do Pará, Escola de Enfermagem Magalhães Barata, Belém, PA, Brazil.

**Keywords:** Child, Adolescent, Quality of Life, Transsexualism, Transgender Persons, Gender Identity, Adolescente, Qualidade de Vida, Transexualidade, Pessoas Transgênero, Identidade de Gênero, Niño, Adolescente, Cualidad de Vida, Trassexualismo, Personas Transgénero, Identidad de Género

## Abstract

**Objective:**

to describe attributes associated with the Quality of Life of Brazilian transgender children and adolescents according to their own perception.

**Method:**

descriptive study conducted with 32 participants between eight and 18 years old, who were either interviewed or participated in focus groups. The statements were transcribed, grouped with the aid of the *Interface de R pour les Analyses Multidimensionnelles de Textes et de Questionnaires* software, version 0.7 alpha 2 and described according to the definition of Quality of Life by the World Health Organization concerning to the mental, physical, and social dimensions.

**Results:**

it was possible to identify the family nucleus as the main social support for transgender children and adolescents. However, the experience of prejudice and discrimination were negative attributes associated with Quality of Life.

**Conclusion:**

the statements indicate that lives of transgender children and adolescents are impacted by social, physical, and mental factors due to the stigma and discrimination experienced. It is expected to contribute to the formulation of public policies related to transgender children and adolescents and expand the discussion on the citizens’ duties and rights in relation to transsexuality.

## Abstract (pt)

**Objetivo:**

descrever atributos associados à Qualidade de Vida de crianças e adolescentes transgêneros brasileiros segundo sua própria percepção.

**Método:**

estudo descritivo, realizado com 32 participantes entre oito e 18 anos, entrevistados ou que participaram de grupos focais. Os depoimentos foram transcritos, agrupados com auxílio do *software Interface de R pour les Analyses Multidimensionnelles de Textes et de Questionnaires*, versão 0.7 alpha 2 e descritos segundo a definição da Qualidade de Vida pela Organização Mundial da Saúde no que concerne às dimensões mental, física e social.

**Resultados:**

foi possível identificar o núcleo familiar como o principal meio de suporte social das crianças e adolescentes transgêneros. Entretanto, a vivência de preconceito e discriminação foram atributos negativos associados à Qualidade de Vida.

**Conclusão:**

Los depoimentos indicam que a vida das crianças e adolescentes transgêneros é impactada por fatores sociais, físicos e mentais em virtude do estigma e discriminação vivenciados. Espera-se com esse estudo contribuir para a formulação de políticas públicas relacionadas às crianças e adolescentes transgêneros e ampliar a discussão sobre deveres e direitos dos cidadãos frente à transexualidade.

## Abstract (es)

**Objetivo:**

describir los atributos asociados a la Calidad de Vida de niños y adolescentes transgénero brasileños, de acuerdo con su percepción.

**Método:**

estudio descriptivo, realizado con 32 participantes entre 8 y 18 años, entrevistados o que participaron en grupos focales. Los testimonios se transcribieron y clasificaron con el soporte del *software Interface de R pour les Analyses Multidimensionnelles de Textes et de Questionnaires*, versão 0.7 alpha 2 y fueron descriptos de acuerdo con la definición de Calidad de Vida de la Organización Mundial de la Salud en lo concerniente a las dimensiones mental, física y social.

**Resultados:**

fue posible identificar al núcleo familiar como el principal medio de soporte social para niños y adolescentes transgénero. Sin embargo, haber vivido experiencias de prejuicio y discriminación fueron atributos negativos asociados con la Calidad de Vida.

**Conclusión:**

los testimonios indican que la vida de los niños y adolescentes transgénero se ve afectada por factores sociales, físicos y mentales debido al estigma y la discriminación que experimentan. Se espera que este estudio contribuya a la formulación de políticas públicas relacionadas con los niños y adolescentes transgénero y amplíe la discusión sobre los deberes y derechos de los ciudadanos en relación a la transexualidad.

## Introduction

Transgender individuals are those whose gender identity differs from their biological sex^(^ 1 ^)^. The so-called transgender persons are those who socially claim to be recognized as women, men, or as non-binary gender^(^ 2 ^)^.

The diagnosis of transsexualism first appeared in 1975^(^ 3 ^)^. In 1980, in the Diagnostic and Statistical Manual of Mental Disorders (DSM-3), transsexualism is described as a psychosocial condition, defined as “gender identity disorder”^(^ 4 ^)^. In 1990, in the International Classification of Diseases (ICD-10), the terms “transsexualism” (F64.0) and “gender identity disorder of childhood” (F64.2) were used to diagnose individuals who have an incongruity with their biological sex^(^ 5 ^)^. After reviews in the DSM, in its 5^th^edition (DSM-5), the term “gender dysphoria” is used to diagnose individuals who do not identify with their assigned gender^(^ 6 ^)^.

In the ICD-11 version, the diagnosis of transsexualism was removed from Chapter V (F00-F99) on mental and behavioral disorders. Chapter 17, concerning the conditions related to sexual health, the term “gender incongruence” was created and included (HA60, HA61, HA6Z)^(^ 7 ^)^.

The effort to depathologize trans-identities has been claimed by movements of political struggle of transgender individuals, being recognized by social studies of gender, of sexuality, and by the World Health Organization (WHO). This claim starts from the premise that transgenderity is not configured as a disease, but as another possibility of expression and experience of non-cisgender people^(^ 8 ^-^ 9 ^)^.

Transgender individuals recognize their gender identity even in childhood, and often express this desire by adopting symbolic elements of this gender^(^ 10 ^-^ 11 ^)^. Children between 17 and 21 months of age learn to label themselves as boys or girls, this becoming more noticeable around 2 years old. Gender identity occurs gradually, starting between 2 and 3 years old. Between 6 and 7 years old, children are aware that their gender will remain for life^(^ 10 ^-^ 11 ^)^.

Children who do not identify with their assigned gender confront difficulties of social oppression and experience feelings of preconception, social discrimination, and denial about their own gender identity, a fact that makes self-acceptance a suffered process^(^ 11 ^)^. Feelings experienced during childhood and adolescence can cause psychosocial damage until adulthood, and they may last. These periods are marked by the beginning of the construction of identities, experiments, discoveries, social affirmation, and questions related to gender, having as main reference the way in which the body appears in society and behavioral patterns to be performed by boys and girls^(^ 12 ^-^ 13 ^)^.

The socialization process begins in the family, in the so-called Primary Socialization. It is in the family that the individual learns rules, personal values, and to interact with the world, therefore being the primordial socialization for the formation of the individual. Secondary Socialization is understood as the experience in the social world - school, work, group of friends - and this is constantly changing, since society is not immutable^(^ 14 ^-^ 15 ^)^.

In the field of health, Quality of Life (QoL) represents a multidimensional construct, with applicability and relevance for people of all age groups, cultures, geographic location or socioeconomic situation^(^ 16 ^)^.

The relevance of studies that address the QoL of children and adolescents is highly recognized^(^ 17 ^)^, as there are numerous factors that can influence their perception^(^ 18 ^)^. Understanding and knowing the QoL perspective of healthy children and adolescents is important for the development of public policies that promote their health and well-being^(^ 19 ^-^ 20 ^)^.

The WHO defines that “quality of life is the individual’s perception of their position in life in the context of the culture and value systems in which they live and in relation to their goals, expectations, standards and concerns”^(^ 18 ^,^ 21 ^-^ 23 ^)^.

The QoL of transgender children and adolescents is mostly lower when compared with cisgender children^(^ 24 ^)^. However, there are few studies addressing the QoL of transgender children and adolescents^(^ 25 ^)^.

This study aimed to describe the attributes associated with the QoL of Brazilian transgender children and adolescents, according to their perception. It was conducted aiming to provide subsidies to parents or caregivers, family members and people belonging to the secondary socialization nucleus such as teachers, other children or adolescents, health professionals, among others, that enable support at this stage, with a view to reducing psychological, physical and social suffering in this population.

## Method

A descriptive study with a qualitative approach. Data collection was conducted through Focus Group (FG) and semi-structured interviews, developed at the Transdiciplinary Ambulatory of Gender Identity and Sexual Orientation (AMTIGOS) of the Institute of Psychiatry, Hospital das Clínicas, Faculty of Medicine, University of São Paulo (IpqHC - USP/SP). In the outpatient clinic, the interventions for transgender children and adolescents are focused on psychosocial care, including the family in this process. Psychotherapeutic care is provided for children and adolescents and their families.

The option to include interviews in the data collection was due to the difficulty in finding participants from the studied population, mainly children. The interviews were conducted among August to November 2018, with Brazilian transgender children and adolescents, between eight and 18 years old.

The invitation to participate in the study was made at the end of the Parents Group. Some reported interest in participation, but with an unfeasible time to stay.

The FGs and interviews started with a brief presentation of the moderator and the study. One adolescent felt uncomfortable at one point and left the room, returning after a few minutes and a child withdrew it is Assent before the group started.

The questions compose the Interview Focus Group questionnaire proposed by the DISABKIDS^®^ group and adapted for the study by the Research Group on Health Measures (*Grupo de Pesquisa sobre Medidas em Saúde*, GPEMSA) - The National Council for Scientific and Technological Development (CNPq in Portuguese), University of São Paulo at Ribeirão Preto College of Nursing (EERP - USP in Portuguese).

Children and adolescents with no ability to understand the questions were excluded. No specific instrument was used to measure it, which was done through observation by the researcher and/or medical or parents/caregivers reports.

The FG technique allows for social interaction among the participants, who commence to consider each other’s opinions to formulate answers and ideas, as well as enable a trusting relationship with the moderator. Another positive factor of the FG is sharing and exchanging common experiences among the participants^(^ 26 ^)^.

The activities were recorded, the textual materials obtained were fully transcribed, organized into two *corpus*(interview transcripts), one for children and the other for adolescents, submmited to Descending Hierarchical Classification (DHC) analysis supported by *Interface de R pour les Analyses Multidimensionnelles de Textes et de Questionnaires*(IRaMuTeq) software, version 0.7 alpha 2^(^ 27 ^)^.

The classes were defined by the words that were most associated with it due to the average frequency of occurrences. Since the adolescents had longer segments of speech, the values of statistical significance for the inclusion of words were α = 0.05 for children and α = 0.0001 for adolescents, respectively. The categories resulting from the statements were described according to the WHO definition concerning to the mental, physical, and social dimensions^(^ 21 ^)^ and according to Primary and Secondary Socialization^(^ 14 ^-^ 15 ^)^.

This research was approved with regard to the ethical aspects, in accordance with Resolution of the National Health Council 466/2012 (CAAE: 87039918.3.0000.5393).

## Results

12 children and 20 adolescents participated in the study. Three FGs were conducted, one with 10 children and two with 13 and 10 adolescents, respectively. In the second FG, three adolescents who had been in the first one participated, and two children were interviewed, as there were not enough participants for an FG^(^ 26 ^)^. Both FG of children lasted 60 minutes and those of adolescents lasted 90 minutes.

The children’s average age was 9.9 years old, with an SD of 0.9 years old (values between eight and 11 years old). In the adolescents, the average age was 15.8 years old, with an SD of 1.6 years old (values between 13 and 18 years old). Regarding gender, 58.3% of the children identify themselves as females and, in the group of the adolescents, 80.0% identify themselves as males.

Due to the specificity of the groups studied and to the confidentiality of the children and adolescents, it was decided not to present the statements.

**Figure 1:**
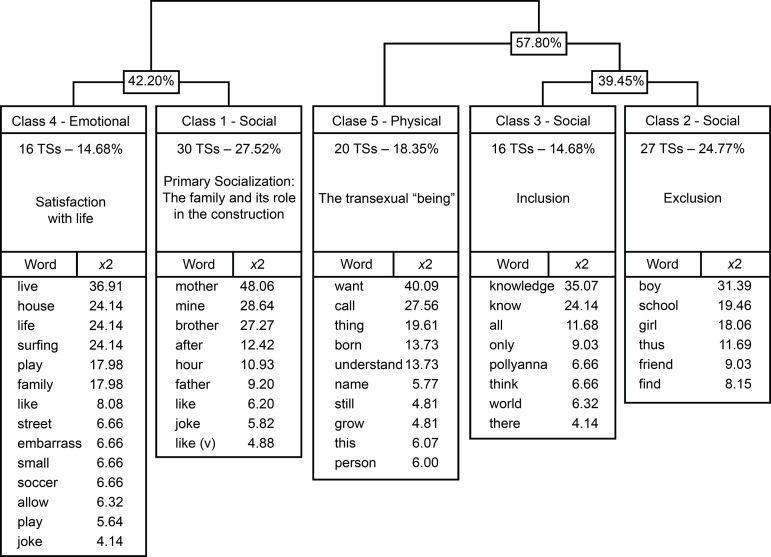
Dendrogram of the Descending Hierarchical Classification of the *corpus* “Transsexuality and childhood: Aspects of quality of life”

Regarding the children’s *corpus*, it was composed of 111 numbers of texts (number of statements), which were divided into 149 text segments (TSs). 3,559 occurrences were analyzed (total number of words contained in the *corpus*), resulting in a mean of 23.88 occurrences *per* segment. The DHC used 73.15% (109) of the text segments, classified into five classes (Figure 1).

### Class 1 (27.52%): Primary Socialization: The family and its role in the construction of identities

The elements present in this class represent the role of the family as the idea of primary socialization that occurs in childhood, related to the child’s emotional aspects. The most frequent words - *mother, my, brother, father, like and play* - refer to everything the children mention they like in life, with the mother being the most influential figure. In contrast, the father is portrayed in two ways: as a present personality, of pride; or, on the other hand, absent. According to reports, the father is also the one who presents the greatest difficulties in terms of accepting transgenderity in the family.

### Class 4 (14.68%): Emotional: Satisfaction with life

The content of Class 4 is associated with Class 1, with the family nucleus very present. The statements demonstrate that transsexuality does not affect children’s activities, style and daily life. They mention leisure activities such as *surfing, playing, beach, soccer*.

No negative attributes were observed in this class.

### Classes 2 and 3 (39.45%): Social Inclusion and Exclusion

The social dimensions become evident in these classes, in which transgender children begin to perceive themselves in the surroundings as different or not, depending on the experience of each one. The words *boy, school, girl, friend* elucidate several aspects, such as: discovery of identity, acceptance of the other, and discrimination at school, this being a phase in which the children begin the process of leaving the family unit for social interaction.

The respect of friends and family is extremely important, as it protects mental health, and provides the physical and social well-being of transgender children and adolescents. For them, social recognition of their transgender identity leads to positive influences on QoL and provides greater psychological comfort in the process of building the identity of these children, who are in a discovery process.

The school environment was identified as the space of social coexistence where children experience situations of discrimination, preconception, and exclusion, corroborating the social stigmatization of trans-identity. As a protective factor, they prefer not to mention transgenderity for fear of experiencing these feelings, considering that many have already experienced them. Peer acceptance can directly influence the QoL of transgender children and it is important that they feel free from stigma and discrimination.

### Class 5 (18.35%): Physical: The transsexual “being”

This class includes issues such as the social name and their desire to be recognized with the gender that represents them, being treated by the name they have chosen and by pronouns in the feminine or masculine - she/he, for example - according to their gender identification. The social name is important for both transgender children and adolescents because it is chosen by them and represents their true gender identity. There are also questions about the body and how self-image is perceived by these children. The desire for change is evident in the statements, which clearly speak about “wanting to have been born a girl/boy”, and they mention the sex reassignment surgery.

For children, the most representative figures in classes 1 and 4, *Primary Socialization* (27.52%) and *Satisfaction with life* (14.68%) are mother, father, and siblings. The children mention their parents when asked about “what do you like most in your life?” and, contradictorily, when asked about “what makes them sad”, they also mention the difficulty of acceptance and respect at home, with the father figure being cited the most. Thus, the focus remains on the family level, that is *in Primary Socialization*.

**Figure 2:**
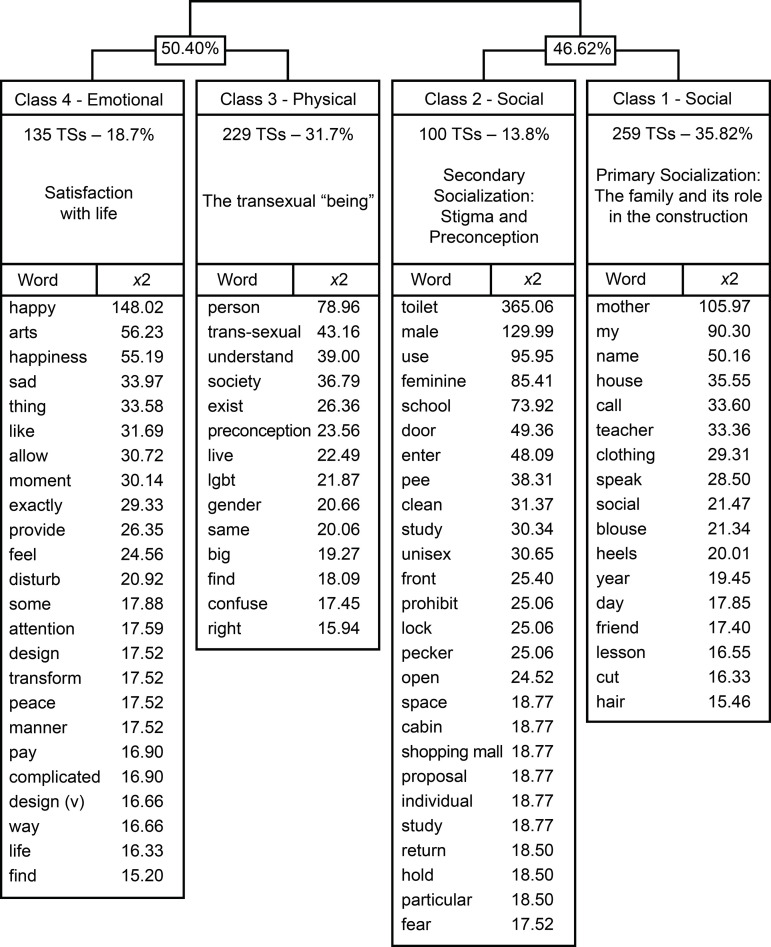
Dendrogram of the Descending Hierarchical Classification of the *corpus* “Adolescence and transsexuality: Aspects of quality of life”

The adolescents’ *corpus* was composed of 402 numbers of texts, resulting into 769 text segments (TSs). 21,613 occurrences were analyzed, resulting in an average of 28.10 occurrences *per* segment. The words considered in the analysis showed chi-square values equal or greater than 15.14 (p ≤ 0.0001 for DF = 1). The DHC used 94.02% (723) of the text segments, classified into four classes (Figure 2).

### Class 1 (35.82%). Primary Socialization: The family and it is role in the construction of identities

This class represents the role of family support in their lives. The word “mother” is the most representative figure in this experience. The mother - confidant - is the first person whom adolescents feel secure to tell about their trans-identity; they are the ones who support them during the puberty period and to whom they always resort in search of psychosocial support and “approval”.

Acceptance inside their family represents important security and emotional support for coping with experiences of violence, preconception, and discrimination reported during the FGs. They also report wearing their mother’s clothes and shoes during the discovery period. The words *cut* and *hair* portray a moment of great significance in the lives of adolescents, when the transformation with family support, usually from the mother, is a positive milestone. The words *name, home, calling, teacher, speaking, social* express the adolescents’ desire to be recognized by their social name. The statements refer to the difficulties faced by the participants so that people respect and make use of the social name (linked to the use of treatment pronouns according to the gender) and the impact that this represents on family, school, mental health, and QoL.

The teacher is recognized as an important figure in this process, being often cited by the adolescents in the student-teacher relationship. The attendance list system used in the classroom is often mentioned, as adolescents feel embarrassed when called out loud by the registration name, which is not representative of their trans-identity, and some teachers do not always respect the social name. In contrast, in some schools, the teachers show respect for the students’ self-identification. The family environment and the mother are reported as important support, in all these situations.

There are also cases in which the mother contradicts the adolescents’ wishes, making them wear clothes or accessories used by the gender that does not represent them, causing mental suffering and social isolation.

### Class 2 (13.80%). Secondary socialization: Stigma and Preconception

This class complements the previous one, but transcends the difficulties and preconceptions experienced in society, with the school setting as the main space for social manifestation. Thus, the stigma in social life seems to be greater when compared to the family environment, once the family appears as a support base.

Usually representing an obstacle the use of public toilets was one of the main problems reported in the statements. The difficulties imposed by the schools lead to emotional, psychological, and health harms, as there is an obstacle for the adolescents to use the school toilet they consider appropriate for their gender and they spend the entire period without going to the restroom, which can result in harms to health, like urinary infections. Fear of aggression (physical, verbal, and non-verbal) and harassment when using public toilets, is another problem faced by them.

### Class 3 (31.7%). Physical: The transsexual “being”

The speeches in this class elucidate the discoveries about being a transsexual and the physical difficulties of adolescents regarding their gender self-designation. Issues are often raised about their own body, and there is a strong desire for transformation through sex reassignment surgery and hormone therapy. This class also shows the fragility that permeates the intimacy of transsexual individuals, as well as the difficulty of their own acceptance.

The adolescents report the importance of information and conversation, the desire to be noticed, discussed, and understood as individuals who are part of society, and do not feel excluded. The fact that they are respected by peers may directly influence their QoL, which allows for a feeling distant from stigma and discrimination.

Sometimes they express anguish in their relationships in society and declare that people are unaware and do not seek to understand what it is to be a transgender person; in addition, they are labeled as “the transsexual person”, which causes discomfort. In this perspective, other people’s curiosity and intimate questions about the body often bring discomfort to the adolescents, as they feel their intimacy exposed and invaded, making them feel strange or abnormal.

### Class 4 (18.7%). Emotional Satisfaction with life

The speech in the text excerpts of this class is characterized by the predominance of statements that refer to happiness and well-being, with art, leisure activities, and sharing moments with family and friends being frequently cited.

Another fact to consider refers to the words *happiness* and *happy*, which are entirely linked to statements that portray the adolescents’ joy when they feel the freedom to express themselves as they are, reflecting self-acceptance and respect of the other in relation to them. Furthermore, knowing that they are fighting for a cause reflects happiness, as they feel that they can transform the suffering they experience into joy for others in the future.

## Discussion

In a study about QoL^(^ 16 ^)^ conducted with children and adolescents were found significant differences in the dimensions studies when analyzing the two age groups, and, therefore, it was decided to use the analysis of the results separately for the two age groups: children (eight to 12 years old) and adolescents (13 to 18 years old).

For both children and adolescents, the results demonstrate that the family nucleus is the main social means of reference and coexistence.

### Primary socialization and Satisfaction with life

Gender identity is built throughout life, according to sociocultural experiences and influences, being a complex and constantly changing event^(^ 14 ^-^ 15 ^)^. When identity construction begins, family is the first social means of coexistence during childhood, characterized as primary socialization^(^ 14 ^-^ 15 ^)^. In the family environment, the initial connections are formed that guarantee the child’s ability to relate and develop affective bonds later in life. In this way, family support is an important element for the healthy process of gender self-designation.

A study that used the FG technique to develop the *KIDSCREEN* questionnaire to measure the QoL of healthy children and adolescents concluded that the most important aspect in the QoL of the group of children was associated with family relationships. However, for the adolescents, the aspects considered most important for QoL raised in the FGs were social relationships^(^ 28 ^)^.

Accordingly, in the case of the adolescents, this construction in the *Primary Socialization* class (35.82%), although still significant, begins to change its axis and the adolescent begins to experience new identifications, a moment in which he/she seeks to understand him/herself in the world and is faced with decision making. Therefore, class 4, *Satisfaction with life*(18.7%), is not exclusively related to the family, although this is still present in the statements of the adolescents, it appears as an emotional support to deal with external issues. The adolescent does not cite the family as the main coexistence nucleus, and there are more emerged issues related to the school, to the use of public places, to the places for sociability, and to the social knowledge about transgender/transsexuality, among other aspects. Such results are corroborated in other studies^(^ 14 ^-^ 15 ^)^.

Positive relationships with family members, close friends, co-workers or the school function as a social support network for building the social identity of transgender individuals, with the family environment being the main source of this support^(^ 29 ^-^ 31 ^)^.

A survey conducted with transgender people in two Brazilian states, which investigated the impact of parental support on the risk factors in the process of self-designation of transgender people, of 421 respondents 29.45% (124) reported not having parental support during gender self-designation, 20.43% (86) received less support, and 20.43% (86) said they received support from their parents. When asked about the need to move away from the family because they are transsexuals, 40% answered that this was an experienced reality^(^ 30 ^)^.

Consistent with the results of previous studies, the authors claim that family support in childhood brings benefits to the lives of transgender people in adulthood, as the family is a direct support for children and adolescents during the growth and development period, in particular, in their sexuality and gender self-designation process^(^ 31 ^-^ 33 ^)^.

Accordingly, the authors portray the importance that family support represents in the lives of transsexual people and conclude that intrafamily discrimination, as the lack of emotional support, represent a risk to the mental health and QoL of these individuals. Therefore, family support is directly associated with the QoL of transgender people^(^ 34 ^-^ 35 ^)^. Thus, the importance is justified of interventions in different social sectors, particularly in the family health care services, so that they can act as facilitators of the acceptance and understanding by parents and/or caregivers, in order not to reject or discriminate transgender children.

### Secondary socialization: social inclusion and exclusion

For the adolescents, the assessments associated with class 2, *Secondary socialization*, were fewer (13.8%) in relation to those of classes 2 and 3 of the children (39.45%), which also correspond to *Secondary socialization*. For the children, in addition to social exclusion, mostly signaled by the adolescents, acceptance by peers is something important because, in this phase, the processes of greater autonomy in the school setting are accentuated, in which they begin to experience situations of preconception and exclusions, when they leave the more protected space: the family nucleus. Therefore, there are particularities in the way of experiencing these situations among children and adolescents.

The school setting becomes an environment that increases the vulnerability of transgender students, since they feel insecure in these places due to their sexual orientation and/or gender expression and identity, being targets of verbal, symbolic, physical, and discriminatory violence on the part of school peers and staff^(^ 32 ^,^ 36 ^-^ 38 ^)^.

Another issue related to the children, in addition to the expectation of acceptance by peers for inclusion, is the desire to be recognized by the gender they identify themselves with.

However, as mentioned, adolescents feel this socialization in another way, perceiving school and public environments, among others, mainly as barriers to their social inclusion. In addition, the fear of being attacked in public spaces is constant. According to the *Trans Murder Monitoring Project*, the number of reported homicides of transgender people has been increasing each year, with Brazil being the country that kills the most transgender people in the world^(^ 39 ^)^.

A research study confirmed that, of 7,989 Lesbian, Gays, Bisexual, Transgender and Queer (LGBTQ) students, more than half (55.2%) suffered verbal violence and 11.4% physical violence due to their gender expression. In this survey, 33.1% of the students reported hearing negative comments about transgender people^(^ 36 ^)^.

Corroborating the results of this research, attention is drawn to the daily violence that is disguised and naturalized in relation to the gender identity of transgender people^(^ 40 ^-^ 41 ^)^. To exemplify, there is the use of the toilets built according to the binary sexual model - male/female - which represents a constant inadequacy for transgender individuals, who are often inhibited, embarrassed or even forbidden to use them for not fitting these cisheteronormative or binary patterns^(^ 40 ^-^ 41 ^)^.

Considering the important role of the school in the socialization of children and adolescents, it is important that issues related to sexual and gender diversity are adequately addressed and discussed in these environments^(^ 42 ^-^ 44 ^)^. For this, it is necessary to prepare teachers and staff to deal with these issues, as there is a tendency towards the biologization of sexuality in the schools. In this sense, the potential of the present study is believed to contribute to such approaches and discussions in different care contexts, understanding that, from an expanded health perspective, the school is also configured as a care space.

Thus, when discussions about sex, gender, and sexuality are proposed in the school/educational setting, the focus is largely on issues of Sexually Transmitted Infections (STIs), means of prevention, reproductive functions, body physiology, non-desired pregnancy, contraceptive methods, among others. Consequently, sex education is reduced to reproductive and biological functions, leaving aside the associated historical, subjective, political, and socio-cultural aspects^(^ 36 ^,^ 42 ^-^ 43 ^,^ 45 ^)^.

Such actions are reinforced because, due to the prejudice experienced by transgender students in the schools^(^ 36 ^,^ 46 ^)^, there is a high dropout rate and few of these students advance to higher education, compromising their academic performance and their exercise of citizenship^(^ 47 ^)^.

Therefore, positive supportive relationships allow transgender children and adolescents to deal effectively with discrimination and face a social system full of challenges^(^ 32 ^,^ 48 ^)^. However, when talking about transsexuality, support networks and social relationships are weakened and demarcated by prejudice and stigma^(^ 49 ^)^ and by institutionalized transphobia (fear, aversion, and exclusion to trans-existence)^(^ 9 ^)^. When faced by these people, such situations lead to a low QoL, as they result in individual and social representations of negative (self)image, in a feeling of inferiority in relation to the other, in loneliness, psychological suffering, depression, and suicide attempts (or concretization)^(^ 34 ^)^.

### Physical Attributes: the transsexual “being”

It appears that the physical aspects are more accentuated in adolescence, class 3 (31.7%) in relation to children, class 5 (18.35%) in the “*The transsexual ‘being’*” class. Adolescents, unlike children, have issues regarding self-acceptance, dealing with the body with which they does not identify (difficulty in looking at the mirror and not recognizing themselves, discomfort with secondary sexual characteristics, desire for hormones, the fact of being transgender, and how being in a body considered to be abnormal to the social “standards” interferes in their interpersonal relationships).

In adolescence, the increase in body dissatisfaction is common, since in this phase secondary sexual characteristics develop, making the transgender youths’ non-identification with their own body more accentuated^(^ 50 ^-^ 52 ^)^.

For some transgender individuals, body changes are issues with specificities and relevance, since dissatisfaction with body image is differentiated due to the need for body-gender adjustments to be more evident^(^ 53 ^)^. Despite not appearing in the statements of this research, the literature review shows studies that point to this issue associated with eating disorders in young transgender people^(^ 51 ^,^ 54 ^-^ 57 ^)^.

In this way, transsexuality represents a significant internal anguish, due to the dissatisfaction resulting from the contradiction between the exterior body and gender identification^(^ 55 ^)^. In a research on body dissatisfaction and transsexuality, more than half of the participants (65%) were involved in diets, 25% reported binge eating, 25% stated purging, and 40% excessive exercise^(^ 55 ^-^ 56 ^)^.

Transgenders have lower levels of satisfaction with body image compared to cisgender people. Both transgender women and men exhibit body dissatisfaction in all spheres, not being directly related to male or female genitalia^(^ 58 ^)^. Although in most cases hormonization or surgery can alleviate this discomfort with the body itself, this modification is not the central solution for the low body image satisfaction experienced by transgender individuals. Therefore, the health professionals have an important role in providing information, education, and support for transgender children and adolescents and their parents or caregivers, to provide support for mental health, nutritional, and well-being issues during the process of gender self-designation and body dissatisfaction^(^ 35 ^,^ 53 ^)^. Another axis to be discussed is associated with situations of discrimination and violence due to physical appearance that lead to an increased feeling of isolation and denied existence, which can affect the physical, emotional, and social well-being of transgender individuals^(^ 32 ^,^ 59 ^)^.

Transgender people are marginalized in society and face difficulties in accessing rights such as the recognition of their transgender identity within the family, in the schools, at work, and in social services/sectors, such as the health service/sector. Furthermore, it is known that opportunities in the job market are scarce when it comes to these people, as there is discrimination by society in relation to their expression and non-cisgender identity^(^ 32 ^,^ 34 ^)^.

Thus, for transgender people, it is essential to recognize their gender identity in society, so they can enjoy the rights and access to education, health, housing, citizenship, and job opportunities, among others, equally to others in the population, with respect and dignity^(^ 59 ^)^.

The non-return to the research scenario, in order to conduct new focus groups with the same children and adolescents with the objective of presenting the results of the analysis of their statements, constitutes a limitation of this study. It is understood that returning to the group of children/adolescents to validate their statements would not be recommended because it is a study that sought subjectivities on a theme considered arid, with a great emotional burden. Performing this validation would imply “sterilizing” this material and running the risk of removing a very important aspect from it, which is precisely spontaneity.

## Conclusion

The results of this research indicated that the lives of transgender children and adolescents are impacted by social, physical, and mental factors, mainly due to the socio-culturally experienced stigma and discrimination. Thus, it was possible to identify the family nucleus as the main means of social support for transgender children and adolescents. On the other hand, mostly, the experience of preconception and discrimination were negative attributes associated with their QoL.

It is expected that this study contributes to the formulation of public policies related to transgender children and adolescents and expand the discussion on the citizens’ duties and rights in relation to transsexuality.

From this starting point, it is expected to provide Brazilian transgender children and adolescents with the freedom they need to bring relevant issues to the agenda and thus improve their QoL.

In this sense, it is important to develop public policies that allow for the safety of transgender individuals in their different stages of life and make the population aware of the fact that discrimination and gender violence occur for the most part, due to lack of information and experience, culminating in the genesis of social preconception.
